# Ethical and Legal Debates on Vaccine Infodemics

**DOI:** 10.7759/cureus.52566

**Published:** 2024-01-19

**Authors:** Ayman Youssef, Luis Ulloa

**Affiliations:** 1 Anesthesiology and Perioperative Medicine, Duke University Medical Center, Durham, USA

**Keywords:** vaccination hesitancy, culture and media, misinformation, debates, mandates, coronavirus pandemic, covid-19 vaccine, covid 19

## Abstract

Over the course of three and a half years, the global toll of coronavirus disease 2019 (COVID-19) has claimed the lives of millions of individuals. Scientific breakthroughs, exemplified by mRNA vaccines, have emerged as crucial tools in saving numerous lives and fortifying our defenses against future pandemics. However, the battle against the virus has been complicated by the dissemination of misleading political and ethical information, resulting in avoidable fatalities. Recognizing this phenomenon, the term 'infodemics' has been coined to denote the proliferation of false or misleading information that hinders effective social responses. Given the historical prevalence of infodemics surrounding vaccinations, this discussion delves into the ongoing ethical and legal deliberations concerning vaccination mandates, an indispensable health intervention in the face of pandemics. Governments bear the responsibility of safeguarding their citizens, acknowledging the social requirements imposed by the collective well-being. The protection of both citizens and healthcare workers becomes paramount, considering the potential risks of infection and mortality associated with individuals refusing vaccination. Historically, governments have played a pivotal role in eradicating pandemics through the implementation of vaccine mandates. However, the contemporary landscape is marked by the infusion of political and misleading misinformation, presenting new challenges. Governments are now confronted with an ethical duty to ensure that citizens possess the necessary information to make informed decisions and safeguard their well-being. While grappling with the realization that extraordinary circumstances demand extraordinary responses, the lessons from past pandemics underscore the imperative of prioritizing public health, especially in the context of the high numbers of casualties worldwide. This discourse explores the ethical and legal dimensions surrounding vaccine mandates, with particular emphasis on their relevance to healthcare workers.

## Introduction and background

Legal and ethical factors are as important as medical resources in saving lives during pandemics. During the 1918 Spanish flu, Philadelphia (Pennsylvania, United States) ignored medical guidelines and authorized the World War I Loan Parade which caused the worst outbreak in America with over 12,000 deaths [1]. A century later, in March 2020, political rallies caused nearly 8,000 coronavirus disease 2019 (COVID-19) deaths in Spain, and political rallies for the 2020 United States presidential election caused nearly a thousand COVID-19 deaths [2]. A common factor is misleading political and ethical information igniting medical mistrust and causing unnecessary deaths [3].

The WHO defined “infodemics” as harmful misleading information that obstructs effective social responses [4]. The two worst infodemics target the most effective methods against pandemics: face masks and vaccinations. This challenge forced the United Nations and CDC to enforce guidelines for healthcare personnel (HCPs) to fight misinformation [4,5]. Being at the front line, Sandra Lindsay, an intensive care nurse in New York, United States, depicted the trust of HCPs in COVID-19 vaccination by being the first person vaccinated live on national television in December 2020. However, by August 2021, many HCPs still refused to be vaccinated and over 40% of nursing home staff, with patients at the highest risk for COVID-19, were not fully vaccinated [6,7].

The outbreak of the Delta severe acute respiratory syndrome coronavirus 2 (SARS-CoV-2) variant and vaccine hesitancy forced leading healthcare organizations to advocate for vaccine mandates. Shortly after, Maine’s Department of Health Services (DHHS) required COVID-19 vaccination for all on-site HCPs, and the Federal Administration required vaccination in all Medicare and Medicaid-certified facilities. The Occupational Safety and Health Administration (OSHA) issued an emergency temporary standard (ETS) requiring vaccinations or weekly testing and masking in businesses with over 100 employees [8,9]. Missouri and Louisiana challenged these decisions, and the United States Supreme Court upheld the vaccination mandate for HCPs but not for businesses. These decisions raised critical legal debates. 

## Review

Vaccination: ethical conundrum

HCPs have the moral responsibility to protect patients against infections and misinformation. This commitment rests on the four fundamental principles of Beauchamp and Childress, including the principles of beneficence and nonmaleficence [10]. Beneficence is the obligation "to act for the benefit of the patient" including preventing conditions that can harm. Nonmaleficence is the "duty to not harm or allow harm to be caused" [11]. Patient protection is the strongest argument for vaccine mandates. Although infection control measurements and protective equipment protect patients, vaccination provides the greatest protection for patients and HCPs inside and outside healthcare facilities. Thus, refusing vaccination exposes patients and others to an unnecessary risk of infection and spreading the epidemic in clinical and social settings. Beyond the ethical considerations, there is a legal liability for causing harm by omission, and the duty to rescue states: “Every person must exercise ordinary care to protect themselves and other people from injury. If a person does not exercise ordinary care, they are negligent.” As vaccination is a necessary care against pandemics, refusing vaccination can be considered negligent behavior contributing to the spread of the pandemic and death.

With over 10 billion doses administered worldwide, including 1.5 billion people with three doses, COVID-19 vaccines have been proven to be safe and effective despite temporary adverse effects in a few recipients. The FDA granted an Emergency Use Authorization (EUA) for Comirnaty (Pfizer-BioNTech COVID-19 vaccine), on December 11, 2020 [12]. The European Union first granted conditional marketing authorization to Comirnaty. In the European Union, after the European Medicines Agency (EMA) evaluates the drug efficacy and safety profiles, the European Commission can issue three types of authorization: EUA, conditional marketing authorization (CMA), and standard marketing authorization (SMA) [13,14]. Conditional marketing authorization is granted in cases where not all the clinical data for a drug required for standard authorization are available but the benefit of placing the drug on the market immediately is considered to outweigh the risks related to the temporary incompleteness of the data. Conditional marketing authorization is valid for one year and includes four requirements: (i) there is a favorable benefit-risk ratio for the drug, (ii) all conditions are in place for the company to provide complete data after authorization, (iii) the treatment meets an unmet medical need, and (iv) the treatment benefit outweighs the risk inherent in the fact that additional data are required.

The FDA granted an EUA for Comirnaty after analyzing the data of 18,801 vaccine and 18,785 placebo recipients aged 16 years and older [12]. Comirnaty is a mRNA vaccine administered in two doses given three weeks apart. Unlike live-attenuated or viral-vectored vaccines, mRNA is non-infectious and without DNA integration. The vaccine was 95% effective in preventing COVID-19 disease with eight cases in the vaccine group and 162 in the placebo group. Approximately 12,000 recipients were followed over six months and the common side effects were pain and redness at the injection site, fatigue, headache, muscle or joint pain, and fever. The FDA post-authorization safety surveillance noted an increased risk of myocarditis and pericarditis, particularly within the seven days following the second dose [15]. However, symptoms resolved quickly suggesting a temporary effect, although some individuals required intensive care. On December 18, 2020, the FDA issued a similar EUA for Spikevax (Moderna's COVID-19 vaccine) after analyzing data from 15,185 vaccines and the same number of placebo recipients in a randomized, placebo-controlled study followed more than two months [16,17]. Common side effects were pain at the injection site or swollen lymph nodes in the arm injected, tiredness, headache, muscle or joint pain, nausea, vomiting, and fever. More people experienced these side effects after the second dose. By January 2022, the overall effectivity preventing COVID-19 infections and hospitalizations was 76% and 81% for the Johnson & Johnson vaccine, 72% and 88% for Spikevax, and 70% and 75% for Comirnaty, respectively [16].

Although HCPs prioritize patient safety, they also have the right of autonomy to make voluntary, informed decisions about their health including refusal of medical interventions. This is the basis for "informed consent" in the physician-patient transaction. Although autonomy is the strongest ethical argument against vaccination, this is an individual value that applies to decisions affecting their health only, and its ethical ground fades when the decisions affect other people. As refusing vaccination jeopardizes the lives of others, the value of autonomy fades because it affects public health. This argument was critical for the vaccine mandates that eradicated smallpox and saved countless lives.

The principle of utility is often used to balance equities and overall benefits, risks, and consequences. Vaccination reduces the risk of COVID-19 infection and death; as such, refusing vaccination risks public health. Nursing homes with staff vaccination rates lower than 75% have a significantly higher prevalence of COVID-19 infections and deaths than those with higher vaccination rates. Increased coverage brings substantial benefits with acceptable consequences, and the safety of patients, HCPs, and communities outweighs the individual interests of a few people. Thus, vaccination produces higher social net benefits than harms considering all concerned parties and competing interests [11]. Under the influential public health ethics framework proposed by Childress et al. [18], all five "justificatory conditions" namely effectiveness, proportionality, necessity, least infringement, and public justification advocate to maximize vaccination. However, vaccination should minimize the transgression of individual rights and accommodate medical exemptions and voluntary decisions such as selecting specific vaccines. In all fairness, HCPs must protect patients and the public, and refusing vaccination fails these responsibilities.

Traditional medical ethics focuses on the individual's health using the four ethical principles set by Beauchamp and Childress: autonomy (respect for individual choice), beneficence (doing good), non-maleficence (avoiding harm), and justice (fairness) [19]. However, public health ethics represents a higher level of organization beyond the scope of traditional ethical frameworks, which becomes inappropriate for justifying public health initiatives [20]. Society requires a social contract and community-level directives with an authority to force members to act according to the social organization as stated by Mill: “The only purpose for which power can rightfully be exercised over any member of a civilized community, against his will, is to prevent harm to others” [21]. Thus, the government must reinforce that citizens, even against their individual choice, must follow the public health initiative to prevent harm to others. For example, forcibly locking a citizen into a sanitarium to prevent the spreading of tuberculosis in the community. Unlike traditional medical ethics, community-level ethics focuses on collective beneficence, nonmaleficence, and justice over individual autonomy [18]. Diekema and Marcuse proposed vaccine mandates are ethically justified if the vaccine benefits the person being vaccinated, minimizes harm to other individuals, is the most effective and least risky method to prevent disease spread [22], and if vaccination benefits outweigh potential burdens. If existing vaccines meet all these criteria, vaccine mandates are ethically justified [23]. Another ethical justification uses the “precautionary principle” proposed by Gostin et al., which asserts that public health professionals must “protect populations against reasonably foreseeable threats, even under conditions of uncertainty. Given the potential costs of inaction, it is the failure to implement preventive measures that require justification” [24]. Thus, despite the uncertainty of the long-term effects of the vaccines, it is the failure to vaccinate an individual is the fact requiring ethical justification to fail public protection for reasonably foreseeable threats [25].

Ethical challenges arise in vaccine mandates for all ages, but more especially mandated for children. The ethical arguments for and against vaccine mandates for children and adults are similar, but there are some nuances. One important consideration is that vaccines are one of the most effective ways to prevent infectious diseases, and they protect people who cannot be vaccinated, such as infants. Indeed, vaccination helps to protect everyone even those that choose not to be vaccinated. The decision for vaccine mandates is complex and must weigh the ethical arguments for and against the mandates, as well as the legal and practical considerations to protect individual rights and public health to ensure they are fair and equitable, and that do not disproportionately impact certain groups of people. Although children are least affected by COVID-19 with very low mortality rates (0.001%), the multisystem inflammatory syndrome in children (MIS-C) is a major concern, and SARS-CoV-2 viruses are continuously mutating increasing the mortality risk in children. The strict lockdown refrained children from going to school and social activities, causing severe psychological impact. Vaccinating children is an alternative and effective way to handle the pandemic and its effects on society [17]. Several studies state that the range of benefits resulting from pediatric vaccination both for the children and the community, implementing the program in a scientific manner with the least financial burden for the families seems to be reasonable and makes it both ethical and moral [17].

The WHO has listed vaccine hesitancy as one of the 10 major threats to global health [26]. Parental hesitancy is equally complex regarding efficacy, effectiveness, and long-term side effects as well as religious beliefs and informed consent [27]. As children are the fastest-growing population and have the highest life expectancy, it is important to ensure that the vaccines do not disturb their growth and development. Though the impact of religious beliefs on vaccine hesitancy has been considerably reduced in the present world, it persists, and vaccine hesitancy appears more common among Evangelical Christians in America [28]. In a survey conducted among 27 European countries, vaccine hesitancy was exhibited by 18% of the total participants (n = 42,583) who prayed daily and 11.9% of the participants who never prayed, showing that COVID-19 vaccine acceptance was higher in the people who never prayed. In Bangladesh, COVID-19 vaccine hesitancy among parents was more prevalent among the Muslim population than the non-Muslim population [17]. A parental refusal of the COVID-19 vaccine does not mean that the parents deny neglecting the child’s health. Parents generally have a positive attitude about vaccinating their children but the majority of them are concerned about the unknown side effects, and people who either themselves or family members were not diagnosed with COVID-19 considered naturopathy a better option [27]. It has been reported that countries such as China, Italy, the United Kingdom, the US, Canada, and Israel have a high rate of parental hesitation in vaccinating their children [29]. The informed consent of parents has created various issues in its practical application. Certain legal systems require the consent of both parents, regardless of whether they are married, cohabiting, or divorced. Sometimes the parents’ decision is against the child’s wishes. In such cases, it is important to consider what is in the best interest of the child. The legal system can also intervene and appoint specialized judges to investigate the case and provide verdicts.

Legal background and argumentation

Vaccine mandates are embedded in the history of the US as General George Washington mandated mass inoculation against smallpox during the American Revolution when Washington required his troops to be inoculated with the smallpox virus in 1777 [30]. The earliest evidence of smallpox dates from around 1500 BC in Egyptian mummies. Smallpox is a fearsome threat with a mortality rate of 10-60% and killing around 400,000 people each year in 18th-century Europe, nearly 10 times as many soldiers as died in battle during the seven-year war [31]. The initial inoculation with the smallpox virus was highly risky and can’t be compared with the safety of the current vaccination. In 1796, English physician Edward Jenner tested the first vaccine against smallpox by injecting children’s pus taken from the bumps of a woman suffering from cowpox, which affects cows but is less virulent in humans but equally effective protection against smallpox disease [32]. As this strategy was safe and effective, countries started to impose mandatory vaccination: the US in 1809, Norway in 1811, and Russia in 1812 [33]. England introduced the first free, universal, and compulsory smallpox vaccination in 1840, making vaccination mandatory for all children up to three months and establishing penalties for non-compliance by 1856. The smallpox outbreaks during the Franco-Prussian War prompted many European countries to introduce compulsory vaccination. In 1958, the eleventh World Health Assembly adopted a strategic plan to eradicate the smallpox virus. On January 1, 1967, the WHO launched the smallpox eradication program leading to the eradication of the virus in 1980. Compulsory vaccination enabled the eradication of the smallpox virus, which is estimated to kill over 500 million people in the last 100 years. Smallpox is one of two infectious diseases to have been eradicated, the other being rinderpest in 2011.

The first US vaccine mandate was against smallpox in 1809. Massachusetts law empowered the Board of Health of cities to enforce mandatory, free vaccinations for adults, if necessary for the safety of the community [34]. By 1827, children in Boston were required by law to show proof of smallpox vaccination to attend school. During the 1901 outbreak, the Cambridge Board of Health, Massachusetts, US, mandated vaccination or revaccination of all its inhabitants and a $5 fine for those who refused. Cambridge pastor Henning Jacobson refused vaccination because "he and his son had bad reactions to earlier vaccinations". Jacobson argued that subjecting him to a fine for refusing vaccination was an invasion of his liberty and that people should be able to object to vaccination. The US Supreme Court ruled in Jacobson v. Massachusetts (1905) with a 7-2 majority that states could “reasonably” infringe upon personal freedoms during a public health crisis by issuing a fine to those who refused vaccination. The case granted health boards and the government the authority to pass laws that restricted individual liberty, including punishment, for endangering public health. Smallpox was estimated to have killed up to 300 million people in the 20th century [35]. In 1967, when the WHO launched the vaccination program to eradicate smallpox, there were around 13 million cases a year, killing every fourth victim and leaving most survivors scarred or blinded [36]. Vaccination eradicated smallpox in 1979. Current vaccination programs require kindergarten children to be vaccinated against up to 15 infectious diseases including polio, measles, and varicella.

There are major legal differences in vaccination mandates for children and adults. In the US, the legal authority for vaccine mandates for children is generally found in state law. Most states have laws that require children to be vaccinated against certain diseases to attend school or daycare. These laws are generally upheld by the courts, as they are seen as a legitimate exercise of the state's police power to protect public health. The legal authority for vaccine mandates for adults is more limited. The federal government does not have the power to mandate vaccines for adults, and most states do not have laws that do so. However, some employers, schools, and other organizations may require adults to be vaccinated as a condition of employment, enrollment, or participation. These vaccine mandates are generally upheld by the courts when they do not violate the individual's rights [17].

Vaccination is the best way to control pandemics, yet, a third of Americans refused vaccination even one year after the FDA approval of COVID-19 vaccines [6]. On August 12, 2021, amid the delta COVID-19 outbreak, Maine DHHS required COVID-19 vaccination of all on-site HCPs, unless they qualified for medical exemptions [37]. Physician Dr. John Does and eight healthcare workers refused vaccination claiming religious objections because the vaccines were produced “using abortion-related materials and cell lines”. The appeal reached the US Supreme Court, which denied the religious challenge in Does v Mills (October 29, 2021) in a 6-3 decision stating that the balancing of equities weighed in favor of Maine’s interest to protect public health [37]. The court concluded that the Maine healthcare mandate is applicable because it applies equally to all religious and philosophical beliefs. At the time of this report, Maine, New York, and Rhode Island vaccine mandates for HCPs lack religious exemptions, and the Supreme Court also rejected several religious challenges to the New York State vaccine mandates. At the federal level, the US Secretary of the Department of Health and Human Services, Xavier Becerra, issued on November 5, 2021, an interim final rule requesting vaccination of all Medicare and Medicaid facility staff. The states of Louisiana and Missouri challenged the mandate and the US Supreme Court ruled Biden v. Missouri and Becerra v. Louisiana (January 13, 2022) in a 5-4 per curiam (a judicial opinion representing the entire court), that “Ensuring that providers take steps to avoid transmitting a dangerous virus to their patients is consistent with the fundamental principle of the medical profession: first, not harm.” The Court upheld that the rule fits within statutory language authorizing the DHHS Secretary to enact rules he “finds necessary in the interest of the health and safety.” 

A lawsuit was filed by 117 Houston Methodist Hospital (Texas, US) employees to prevent institutions from requiring employee vaccination. That case was dismissed by US District Judge Lynn Hughes because “This is not coercion. Every employment includes limits on the worker's behavior in exchange for his remuneration” [38]. This ruling follows from the US Supreme Court precedent upholding vaccine mandates under the argument that individual autonomy is not absolute and can be usurped by the power of the state in the interest of public health and safety [20]. Given that COVID-19 is highly transmissible and lethal, it is, therefore, a serious threat to public health and safety that justifies vaccine mandates. The Houston Methodist judgment highlights the parallel legal and ethical argument supporting vaccine mandates; through frequent exposure to sick patients, unvaccinated employees are at increased risk of contracting COVID-19 and transmitting it to vulnerable populations. At the same time, healthcare entities have ethical and legal obligations to provide a safe environment for staff and patients, including infection control protocols, and mandatory vaccination against preventable infections (e.g., varicella virus, influenza virus) for employees with patient contact. Healthcare employees have the option to either be vaccinated or seek employment elsewhere. This upholds the principle of justice provided the mandate is non-discriminatory and fair [39].

In November 2021, OSHA issued an ETS requiring vaccination or weekly testing and masking in businesses with over 100 employees. OSHA estimated these vaccinations would prevent 250,000 hospitalizations and save over 6,500 lives over six months; those estimates predated the Omicron outbreak. Several states challenged the ETS and the US Supreme Court ruled in National Federation of Independent Business v. Department of Labor (January 13, 2022) by 6-3 per curiam, that OSHA lacked statutory authority to mandate vaccination or masking in employees outside the healthcare system [9]. The Court did not view COVID-19 as an occupational danger because it can be transmitted throughout society and vaccinations “cannot be undone at the end of the workday.” However, the OSHA Act does not specify that hazards must be exclusively occupational, and most workplaces are an occupational risk as workers spend long periods in crowded indoor settings with an increased risk of infection for workers and customers. Without a mandate, companies were hesitant to require worker vaccination fearing lawsuits or staff defections. However, multiple examples show that vaccination protects workers without massive resignations. For instance, United Airlines required worker vaccination and after three months, on January 13, 2022, Chief Executive Officer, Scott Kirby noted that although some 3,000 employees had COVID-19 at the time, no vaccinated workers were hospitalized. Before vaccination, COVID-19 accounted for an average of one worker’s death each week. During the subsequent two months, not a single United employee died from COVID-19 [9]. Another example shows that nursing homes lost over 15% of their workforce during the pandemic before vaccination according to the American Healthcare Association/National Center for Assisted Living. When Indiana University Health mandated vaccinations, only 0.3% of employees resigned over vaccination [6].

The legal implications of vaccine mandates are deep and complicated. In terms of the legalities of vaccine mandates, it is important to understand the scope and rationale of the US Supreme Court rulings (including not just the decision about OSHA mandates), and the other looming problems of varied but often limited legislative action that might support mandatory vaccination in healthcare (and other) settings. Legal debates about vaccination are often technical and rooted in statutory language. Case law that might guide decisions today, notably Jacobson, may also be more limited than some authors seem to suggest (e.g. J Blackman, "The Irrepressible Myth of Jacobson v. Massachusetts", which walks through a much more narrow reading of Jacobson, perhaps a more restrictive interpretation of Jacobson than others, but an important one to consider) [40]. It is also worth noting that there is considerable literature from historians of medicine describing how smallpox vaccines (while generally accepted now) faced sincere and important opposition, sometimes grounded in questions of safety and autonomy (e.g. KL Walloch, The Antivaccine Heresy: Jacobson v. Massachusetts and the Troubled History of Compulsory Vaccination in the US) [41]. Efforts to eradicate smallpox, and to make mandatory vaccination more accepted, unfolded over decades in a process that is messy and contested. This is partly why legislative action can be so important, and is arguably missing today, also highlighting the limitations of judicial review to support or protect policies.”

The US Supreme Court’s decision is still criticized because it appears sectarian to HCPs and curtails the government’s ability to respond to pandemics. Vaccine efficacy rests on herd immunity, and thus vaccination of specific social groups like HCPs is not sufficient [42]. As the Supreme Court minimized the "occupational" risk of COVID-19 because it “can be transmitted throughout society” [43], vaccination mandates for HCPs appear sectarian and insufficient to protect patients, because patients can be infected through relatives, visits, and society. As COVID-19 is a widespread pandemic not confined to hospitals, liability to avoid spreading the pandemic and risking the lives of others shouldn’t apply to HCPs only. School vaccination is one of the most effective policies to control infectious diseases with all US states requiring vaccination at school entry [44]. An emergency concern is whether the states will start unwinding school vaccine mandates. Florida has already announced recommendations against COVID-19 vaccination in healthy children, even though the CDC stated: “Vaccination is the leading public health prevention strategy to end the COVID-19 pandemic [45]. Promoting vaccination can help schools safely return to in-person learning as well as extracurricular activities and sports.” Under the US Constitution’s federal system, the states hold primary public health powers [39]. Like the smallpox vaccination, the states are now imposing their regulatory views on state and private workers. At the time of this report, 19 states mandated COVID-19 vaccination of state employees, whereas 12 states banned such mandates. Likewise, nine states allowed private employers to mandate vaccination, but only Montana and Tennessee banned them [46].

However, pandemics spread widely, and states acting alone are unable to take effective measures without federal regulations. Congress has delegated flexible authority to federal agencies to set safety standards on infectious diseases, and consumer products including food, drugs, tobacco, and motor vehicle safety, as well as chemical, nuclear, and environmental hazards [47]. By contrast, the US Supreme Court limits the authority of federal agencies to impose safety regulations. Justices Breyer, Sotomayor, and Kagan stated that the decision “stymies the Federal Government’s ability to counter unparalleled” threats [48]. Public health agencies such as OSHA, CDC, and FDA have the expertise needed to respond to complex, changing medical and scientific evidence. Unlike the smallpox Massachusetts law empowering health agencies to respond to infectious outbreaks, the US Supreme Court’s rule appears to undermine federal health agencies and give itself an outsized role in regulating health policy with unpredictable consequences in future threats. These consequences are unpredictable under the threats of unreasonable, brazen infodemics against decisions such as face masks. As vaccines are the most effective public health intervention against pandemics [49-51], these ethical and legal debates are instructional for future public health challenges. The specifics of these agencies' statutory mandates matter and are ultimately what is contested legally, and recourse might have less to do with the Supreme Court and more to do with addressing questions of mandatory vaccination (and pandemic response generally) through legislative action.

International perspective to reinforce compulsory vaccination

Public education became a relevant aspect of healthcare not only to ensure that patients can make informed decisions but also to public health. As expected, new social networks played an important role during the pandemic, but they appeared more important in spreading misinformation and mistrust than reliable scientific information. Who would have anticipated a shortage of toilet paper or the political implications of wearing facemasks? The pandemic revealed the detrimental impact of these networks as never before had individuals had this potential to spread misinformation without legal liability. A typical example is the misinformation about the association between vaccination and autism [52]. In 1998, Wakefield et al. published an article about 12 autistic children, reporting that eight of these children had the measles, mumps, and rubella (MMR) vaccine shortly before experiencing developmental delays [52]. Soon after the article’s publication, Wakefield held a press conference where he withdrew his support for the MMR vaccine and theorized that the measles virus in the vaccine caused proteins to leak from the intestines and impaired neuronal development [53]. Despite all the scientific flaws, the article received wide publicity and MMR vaccination rates dropped with parents fearing autism [54]. Multiple epidemiological studies refuted this link and the article was retracted [55,56]. In 2002, a Danish study of more than half a million children (born between 1991 and 1998) showed no link between MMR vaccination and autism. The risk of autism in vaccinated children was 0.92 (95%CI: 0.68-1.24) and the risk of another autistic spectrum disorder was 0.83 (95%CI: 0.65-1.07) [57]. However, children without vaccination had a higher risk of death and encephalitis, and one in 20 had pneumonia. And yet, 25 years later after the Wakefield publication, some parents still refuse children vaccination fearing autism [13].

The ethical consequences of vaccination prompted the debate on optimal policies to reinforce compulsory vaccination. Indonesia, Tajikistan, and Turkmenistan imposed mandatory COVID-19 vaccination for all citizens. In January 2022, Italy was the first European country to impose vaccination but only on those over the age of 50, followed by Greece (for citizens aged over 60), and Austria (for all citizens). Compulsory vaccination is not a new challenge, and strict laws worldwide reinforce children’s immunization to attend school. In most cases, the state requires vaccination for multiple infectious diseases from tetanus, whooping cough, hepatitis B, rubella, mumps, varicella, and poliovirus before attending kindergarten. This is probably the most successful example of compulsory vaccination worldwide based on two fundamental principles. The first principle is the isolation of those individuals refusing vaccination to minimize jeopardizing public health. The second is preventing social benefits for those individuals refusing vaccination in other to establish the personal limitations required for the social organization [58]. Society and elected officials have the primary duty to protect citizens. Like imprisoning to protect citizens and the ethical considerations for HCPs to protect patients, society and governments should be able to isolate individuals jeopardizing the lives of other citizens. Governments must protect citizens (accepting the social requirements) who can become infected, and die, by individuals refusing vaccination. Of note, the social commitment of healthcare workers should be bilateral with society protecting them for their social function. Why should healthcare workers risk their lives for those individuals who don’t want to be treated and refuse medical treatment? Like healthcare facilities requiring vaccination, effective policies must include companies requiring worker vaccination. A new debate surges as to whether this "passport" should be ‘flexible’ to include citizens who can confirm natural immunization after recovering from the infection [59]. It is not the same to have recovered from the infection as to have natural immunization. The response is again scientific confirmation of natural immunization with adequate antibody levels and the risk of re-infection by different strains [60,61]. Countries like Switzerland extended the vaccination cards to those who proved they recovered from the infection [59]. The implementation of the vaccine passport can represent an alternative to compulsory vaccination and a reasonable balance between personal freedom and public risk [59].

People refusing vaccination are accountable for the consequences and must face punitive or criminal charges when linked to infections or casualties of other individuals. Infected patients have equal protection rights and should be entitled to demand protection and compensation if their infection is due to a person refusing vaccination. Financial penalties should be substantial and relate to the income as reported in Switzerland and Finland [62]. Other countries like Italy imposed a fixed fine that led to a social inequity with the wealthy willing to pay the penalty to avoid vaccination [62]. It is an ethical duty to ensure that those individuals refusing vaccination have reliable information about the consequences of their decisions. Unfortunately, misinformation spreads quickly under the shadow of weak provisions. Like the recent penalty of $787 million to news broadcaster Fox News for defamation [63], strong financial penalties shall be imposed on individuals or entities spreading health misinformation affecting public health. Although criminal charges should be avoided, they should not be excluded when refusal of vaccination or misinformation is linked to infections or casualty. In April 2022, the US Justice Department announced criminal charges against 21 defendants in nine federal districts across the United States for alleged participation in fraud schemes including marketers and manufacturers of fake COVID-19 vaccination record cards because vaccination cards carry the federal seal of the CDC [64]. Thus, making, selling, and transferring counterfeit cards with the CDC seal are felonies under federal law, which carries a sentence of up to five years in prison. These penalties also apply to those buying or using counterfeit vaccination cards [64]. In addition to forgery charges, offenders can face filing fraudulent information of federal or state vaccination records and conspiracy charges. For example, in the Northern District of California, three defendants were charged in a scheme to sell homeoprophylaxis immunizations for COVID-19 and falsify COVID-19 vaccination cards [65]. Although it is difficult to accept that extreme circumstances demand extraordinary responses, previous pandemics taught us the need to protect compliant citizens and public health as we are reaching nearly seven million COVID-19 casualties worldwide [66].

The international perspective on compulsory vaccination is complex depending on the country. There is no global law or treaty that mandates compulsory vaccination, but there are international organizations that promote vaccination and support countries in their efforts to achieve high vaccination coverage. The WHO is the leading international authority on public health. The WHO's Global Vaccine Action Plan (GVAP) 2019-2030 sets out a roadmap for achieving universal vaccination coverage and eliminating vaccine-preventable diseases. The GVAP emphasizes the importance of compulsory vaccination to protect public health but recognizes that there is no single approach and that countries should tailor their policies to their specific circumstances [67]. The WHO also supports countries in their efforts to address the social and economic barriers to vaccination. These barriers can include poverty, lack of access to healthcare, and vaccine hesitancy [67]. In addition to the WHO, other international organizations that promote vaccination include the United Nations Children's Fund (UNICEF), the World Bank, and the Global Alliance of Vaccine and Immunization (GAVI) Alliance [68]. UNICEF also supports compulsory vaccination and states that compulsory vaccination is essential for protecting children from preventable diseases [69]. The World Bank has funded vaccination programs in developing countries as an essential investment in public health [70]. The GAVI Alliance is a public-private partnership to improve access to vaccines in developing countries [68]. The debate over compulsory vaccination is likely to continue. However, the international community is committed to ensuring that everyone has the opportunity to be vaccinated and protected from preventable diseases.

## Conclusions

Infectious epidemics directly impact humans as a species and society. Compulsory vaccination has eradicated two major infectious diseases including the smallpox virus and rinderpest saving countless human lives. Although it is difficult to accept that extreme circumstances demand extraordinary responses, previous pandemics taught us the need to ensure public health. In general, vaccine refusals risk the lives of others including those that cannot be vaccinated such as infants. However, compulsory vaccination is a complex topic because it requires a balance between fundamental scientific information to make proper ethical and legal decisions to accommodate individual rights versus public health. The special challenges of COVID-19 have evidenced the critical need for public education for individuals to make proper decisions. The growing scientific literature on the topic concurs with the vast benefits that appear to support compulsory vaccination as a reasonable approach to protecting public health by implementing the program in a scientific manner and taking into consideration possible alternatives to preserve individual rights.
